# Effects of copper and cadmium on development and superoxide dismutase levels in horseshoe crab (*Limulus polyphemus*) embryos

**DOI:** 10.1186/s40064-015-1267-1

**Published:** 2015-09-17

**Authors:** Mary G. Hamilton, Christopher Esposito, Mia Malin, Lucas R. Cusumano, Mark L. Botton

**Affiliations:** Department of Natural Sciences, Fordham University, 113 West 60th Street, New York, NY 10023 USA; New York College of Osteopathic Medicine, Northern Boulevard, P.O. Box 8000, Old Westbury, NY 11568 USA; School of Health Sciences, Quinnipiac University, 275 Mount Carmel Avenue, Hamden, CT 06518 USA; Albert Einstein College of Medicine, 1300 Morris Park Avenue, Bronx, NY 10461 USA

**Keywords:** Horseshoe crab, *Limulus*, Oxidative stress, SOD, Pollution, Copper, Cadmium

## Abstract

Pollution by metals may adversely affect organisms through the generation of reactive oxygen species (ROS). In this study, we examined the sublethal effects of two metals, copper and cadmium, on horseshoe crab (*Limulus polyphemus*) embryos. Exposure to copper or cadmium at concentrations of 0.01–10 mg/L for periods of 4, 8, 16 and 24 h had minimal effect on embryo survival except at 100 mg/L Cu. However, metal-exposed embryos took significantly longer to hatch into first instar (“trilobite”) larvae than seawater controls. Levels of superoxide dismutase (SOD), believed to be important in the response to oxidative stress, were determined by Western blotting. Both the Cu/Zn and Mn cofactor forms of SOD tended to be somewhat elevated in metal-exposed embryos, but the increases were neither dose nor time-dependent. Likewise, SOD enzymatic activity showed no significant differences comparing embryos exposed to metals with seawater controls. We conclude that the protective role of SOD’s against ROS produced in response to metal exposure appears to be limited in horseshoe crab embryos, at least under our experimental conditions.

## Background

Horseshoe crabs (Chelicerata, Xiphosura) are “living fossils” with an ancestry dating to the Late Ordovician Period, approximately 445 million years ago (Rudkin and Young 2009). The reproductive biology of horseshoe crabs is unique among living arthropods, in that they spawn in the intertidal zones of estuaries and use external fertilization. The American horseshoe crab, *Limulus polyphemus* (Linnaeus, 1758) spawns on estuarine beaches ranging from Maine to the Yucatan Peninsula of Mexico (Brockmann and Smith 2009). American horseshoe crabs are commercially valuable for the production of *Limulus* amoebocyte lysate, or LAL, which is used to detect bacterial endotoxin in the pharmaceutical industry, and for bait in eel and whelk fisheries (Shuster et al. 2003).

The evolutionary persistence of the group attests to its ability to survive changing environmental conditions such as temperature and sea level. Moreover, this extraordinary resilience implies that all of their life-history stages must have considerable phenotypic plasticity (Anderson and Shuster 2003; Botton et al. 2010). Horseshoe crabs are found in a variety of sediment types and depths, ranging from intertidal flats to the continental shelf (Botton and Ropes 1987) and they consume a wide variety of prey items, including bivalves, polychaetes, small crustaceans, etc. (Botton and Shuster 2003). Adult horseshoe crabs are eurythermal and euryhaline; likewise, their eggs can successfully develop across a wide range of temperatures and salinities (Jegla and Costlow 1982; Laughlin 1983; Sugita 1988; Ehlinger and Tankersley 2004; Botton et al. 2006a; Greene et al. 2011). Optimal salinities for embryonic development are between 20 and 30 practical salinity units (PSU) (Jegla and Costlow 1982; Laughlin 1983; Sugita 1988) but they are capable of surviving in hyperosmotic (50 and 60 PSU) and hypoosmotic (10 PSU) conditions, albeit with some delay in development (Ehlinger and Tankersley 2004; Greene et al. 2011). Optimal temperatures for embryonic development are between 25 and 30 °C (Jegla and Costlow 1982; Laughlin 1983). Ehlinger and Tankersley (2004) reported that constant temperatures >35 °C were lethal to embryos, but embryos acclimated to 13 or 22 °C had close to 100 % survival when heat shocked for 3 h at temperatures as high as 40 °C (Botton et al. 2006a). Stress proteins, including HSP70 and HSP90, play a role in surviving temperature (Botton et al. 2006a) and osmotic stresses (Greene et al. 2011).

A number of important *Limulus* spawning locations along the Atlantic coast, such as Sandy Hook Bay, New Jersey, Jamaica Bay, New York, and New Haven Harbor, Connecticut are known to be heavily impacted by municipal and/or industrial wastes (Botton et al. 1998a, 2006b; Mattei et al. 2015). Previous studies have shown that early life stages of *Limulus* are highly tolerant of oil (Laughlin and Neff 1977; Strobel and Brenowitz 1981), PCB’s (Neff and Giam 1977), and pesticides (Weis and Ma 1987) in comparison to similar stages in marine crustacea. Similarly, *Limulus* embryos and larvae are highly tolerant of metal pollutants including cadmium, mercury, copper, zinc, and tributyltin (TBT) (Botton et al. 1998a, b; Botton 2000).

An important pathway for metal toxicity is through the generation of reactive oxygen species (ROS) including the superoxide anion (O_2_^−^), singlet oxygen (^1^O_2_), hydrogen peroxide (H_2_O_2_), and the hydroxyl radical (HO^·^) (Lesser 2006; Regoli 2011; Regoli and Giuliani 2014). ROS are produced in normal cellular activities such as aerobic respiration in mitochondria, and they are harmful because they can oxidize lipids, proteins, and DNA (Lesser 2006). ROS damage to proteins is referred to as carbonylation; it occurs as additional carbonyl (CO) groups form within the protein with subsequent alterations of structure and possible loss of function (Lushchak et al. 2011). ROS cause lipid peroxidation which can damage mitochondrial and plasma membranes (Lesser 2006; Lushchak et al. 2011). Consequently, cells possess enzymatic oxidants (such as superoxide dismutase, SOD, catalase, and peroxidases) in addition to cytosolic scavenger molecules including vitamin C (ascorbic acid), vitamin E (tocopherol), glutathione, and carotenoids to prevent damage caused by protein carbonylation and lipid peroxidation (Lesser 2006; Regoli and Giuliani 2014). However, increased levels of ROS can also be generated by a variety of environmental stresses including temperature, hyperoxic or hypoxic conditions, salinity change, and chemical pollutants including metals and various organic compounds (Lushchak 2011). Oxidative stress thus occurs “when the steady-state ROS concentration is transiently or chronically enhanced, disturbing cellular metabolism and its regulation and damaging cellular constituents” (Lushchak 2011, p. 15).

In this study, we examined the responses of horseshoe crab embryos to oxidative stress caused by metals. We hypothesized that exposure of *Limulus* embryos to sublethal concentrations copper or cadmium would cause a delay in development to the trilobite stage and cause oxidative stress, as evidenced by changes in SOD activity. Copper is known to have important metabolic roles, as part of the respiratory pigment hemocyanin and as a co-factor for certain enzymes, whereas cadmium has no known metabolic function (White and Rainbow 1985). We studied two forms of SOD, an important enzymatic component of the antioxidant system. There is a cytosolic form with a Cu/Zn cofactor, and a mitochondrial form with a Mn cofactor, though the exclusivity in cellular location between cytosolic and mitochondrial forms of SOD may not be absolute, as shown by Brouwer et al. (1977). We hypothesized that embryos exhibiting metal-induced oxidative stress could respond by one or both of the following mechanisms. First, an increase in ROS could result in an increase in the specific activity of SOD, i.e. a greater turnover of substrate molecules per unit amount of enzyme. Second, oxidative stress could lead to an upregulation of SOD synthesis, i.e. an increased number of enzyme molecules.

## Results

### Viability and developmental rate

**Fig. 1:**
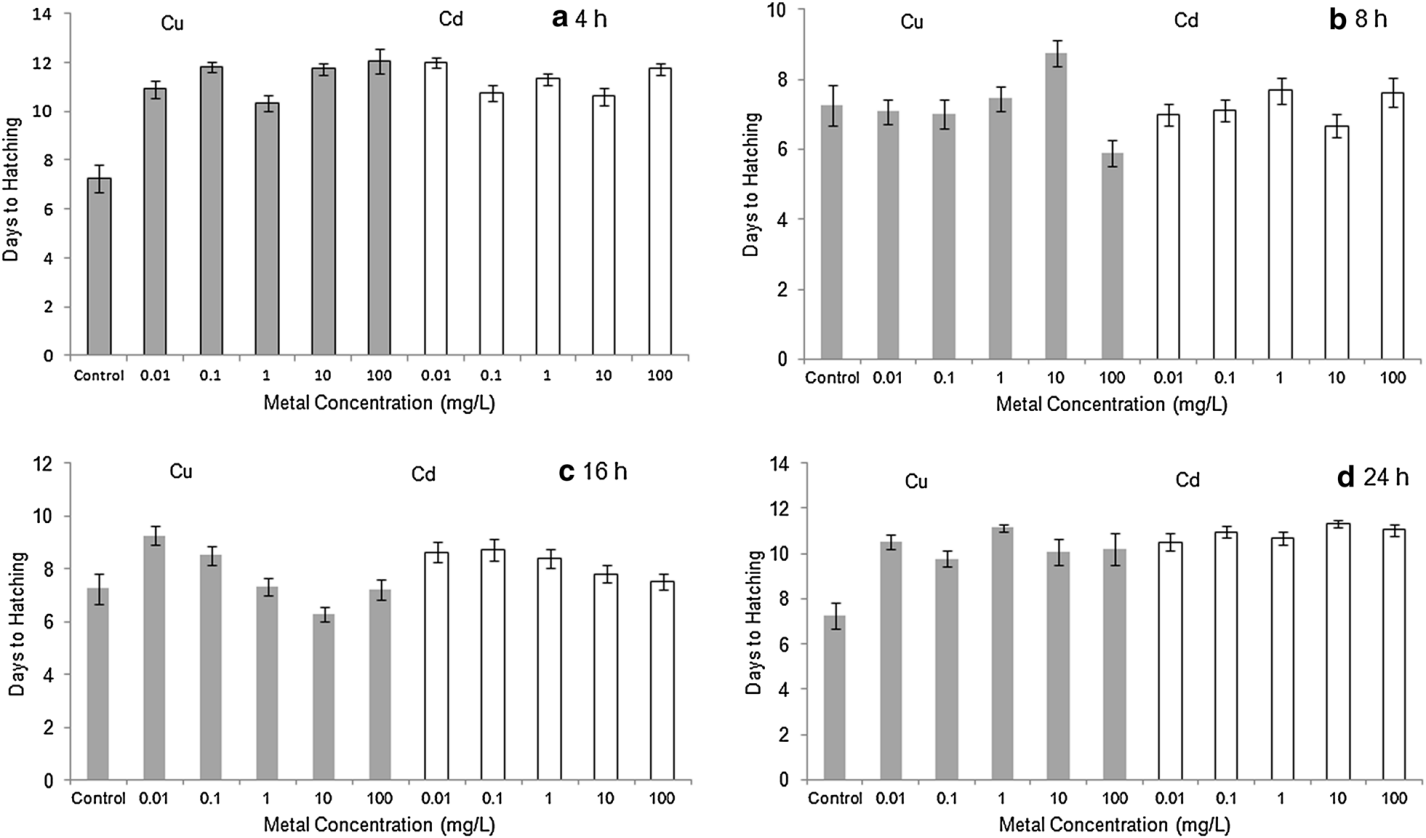
Mean date of hatching (±SE) of Stage 20 *Limulus polyphemus* embryos after **a** 4 h, **b** 8 h, **c** 16 h, and **d** 24 h acute exposure to Cu (*filled bars*) or Cd (*open bars*), followed by transfer to artificial seawater; n = 50 embryos per treatment. Control groups were raised in artificial seawater with no exposure to contaminants

Exposure to CuSO_4_ at concentrations of 0.01–10 mg/L had no detectable effect on embryo survival but there was mortality at 100 mg/L, ranging from 12 % after 8 h exposure to 72 % after 24 h (Table 1). Exposure of embryos to CdCl_2_ at concentrations between 0.01 and 100 mg/L for up to 24 h did not affect survival to the first instar stage. Although mortality was low, hatching was delayed in embryos exposed to Cu or Cd. Control embryos raised in ASW hatched into the first instar in an average of 7.26 ± 0.58 (SE) days, but embryos exposed to metals frequently took an average of 3–4 days longer (Fig. 1). In the 4 and 24 h exposure periods, all metal-exposed groups had significantly slower developmental rates than ASW controls (t tests, *p* < 0.01). In the 8 h exposure group, the Cu 10 mg/L embryos were significantly delayed relative to controls (t test, *p* < 0.04). In the 16 h exposure group, the Cu 0.01 mg/L, Cd 0.01 and Cd 0.1 mg/L embryos were significantly delayed (t tests, *p* < 0.05).

**Table 1:** Proportion of Stage 20-1 horseshoe crab embryos that successfully developed into first instar (trilobite) larvae following exposure to Cu (as CuSO_4_) or Cd (as CdCl_2_), n = 50 embryos per treatment

Time (h)	ASW	Cu concentration (mg/L)					Cd concentration (mg/L)				
		0.01	0.1	1	10	100	0.01	0.1	1	10	100
4	1.00	1.00	1.00	1.00	1.00	0.46	1.00	1.00	1.00	1.00	1.00
8	1.00	0.98	1.00	1.00	1.00	0.88	1.00	1.00	0.98	1.00	0.98
16	1.00	1.00	0.96	1.00	0.96	0.61	1.00	1.00	1.00	1.00	0.96
24	1.00	1.00	1.00	1.00	1.00	0.28	0.96	1.00	1.00	1.00	0.92

*ASW* artificial seawater

### Quantity and activity of SODs

**Fig. 2:**
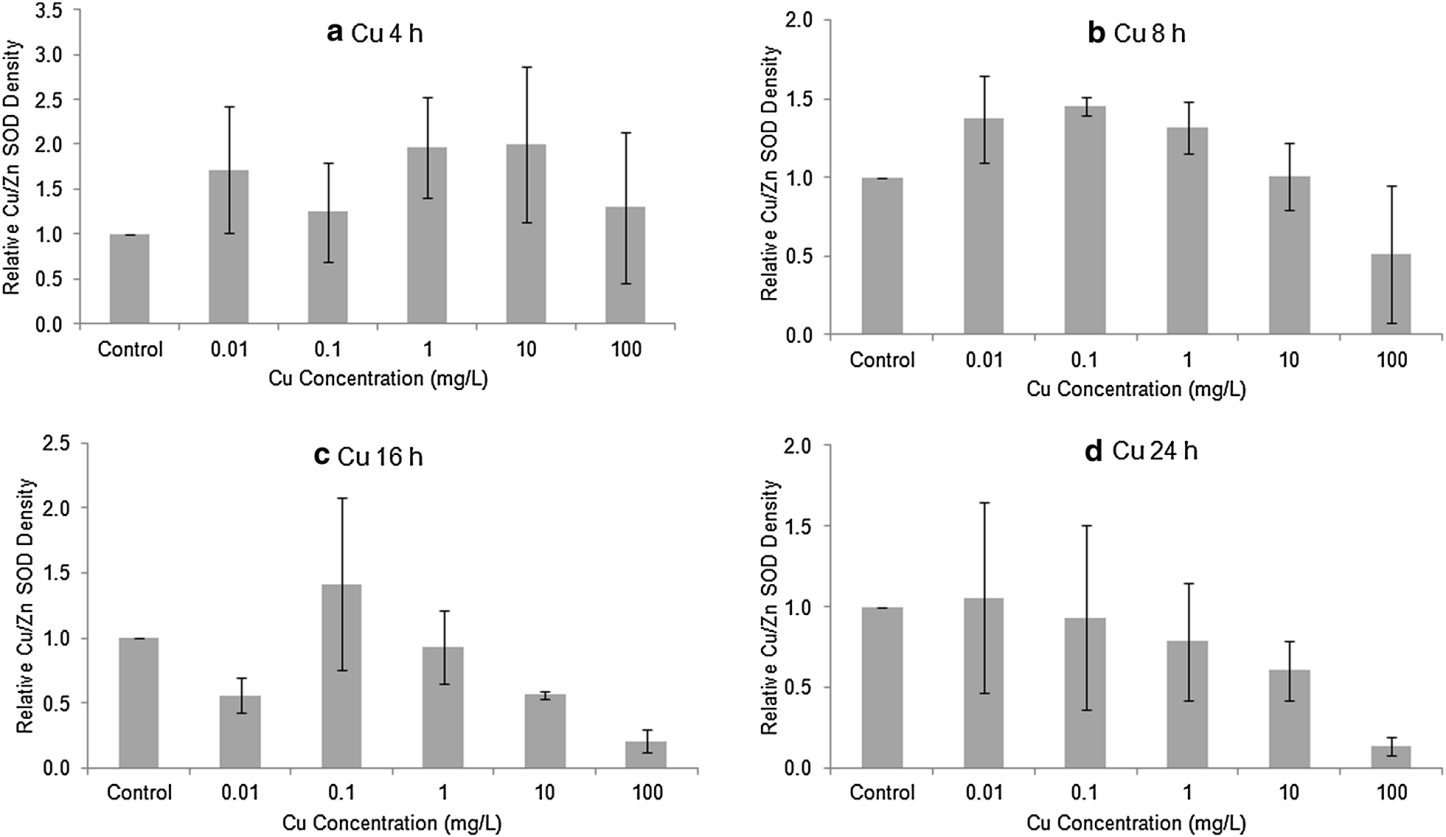
Relative amount of Cu/Zn SOD protein in Stage 20 *Limulus polyphemus* embryos exposed to Cu as determined by Western blotting. Data expressed as the mean ratio (±SE) of CuZn SOD in exposed animals relative to seawater controls (n = 2–4 replicate determinations). **a** 4 h, **b** 8 h, **c** 16 h, and **d** 24 h acute exposure to Cu

**Fig. 3:**
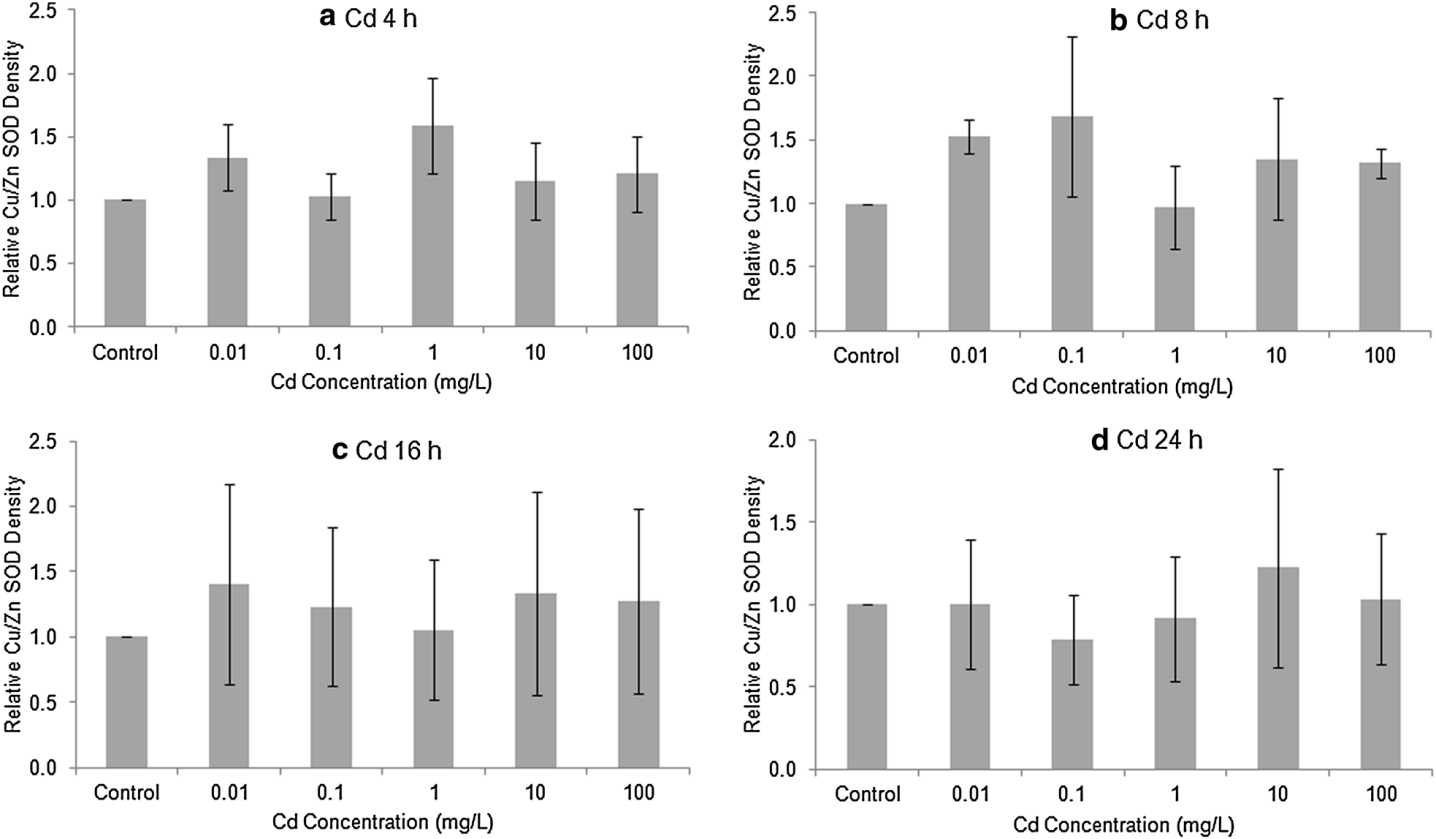
Relative amount of Cu/Zn SOD protein in Stage 20 *Limulus polyphemus* embryos exposed to Cd as determined by Western blotting. Data expressed as the mean ratio (±SE) of CuZn SOD in exposed animals relative to seawater controls (n = 2–4 replicate determinations). **a** 4 h, **b** 8 h, **c** 16 h, and **d** 24 h acute exposure to Cd

**Fig. 4:**
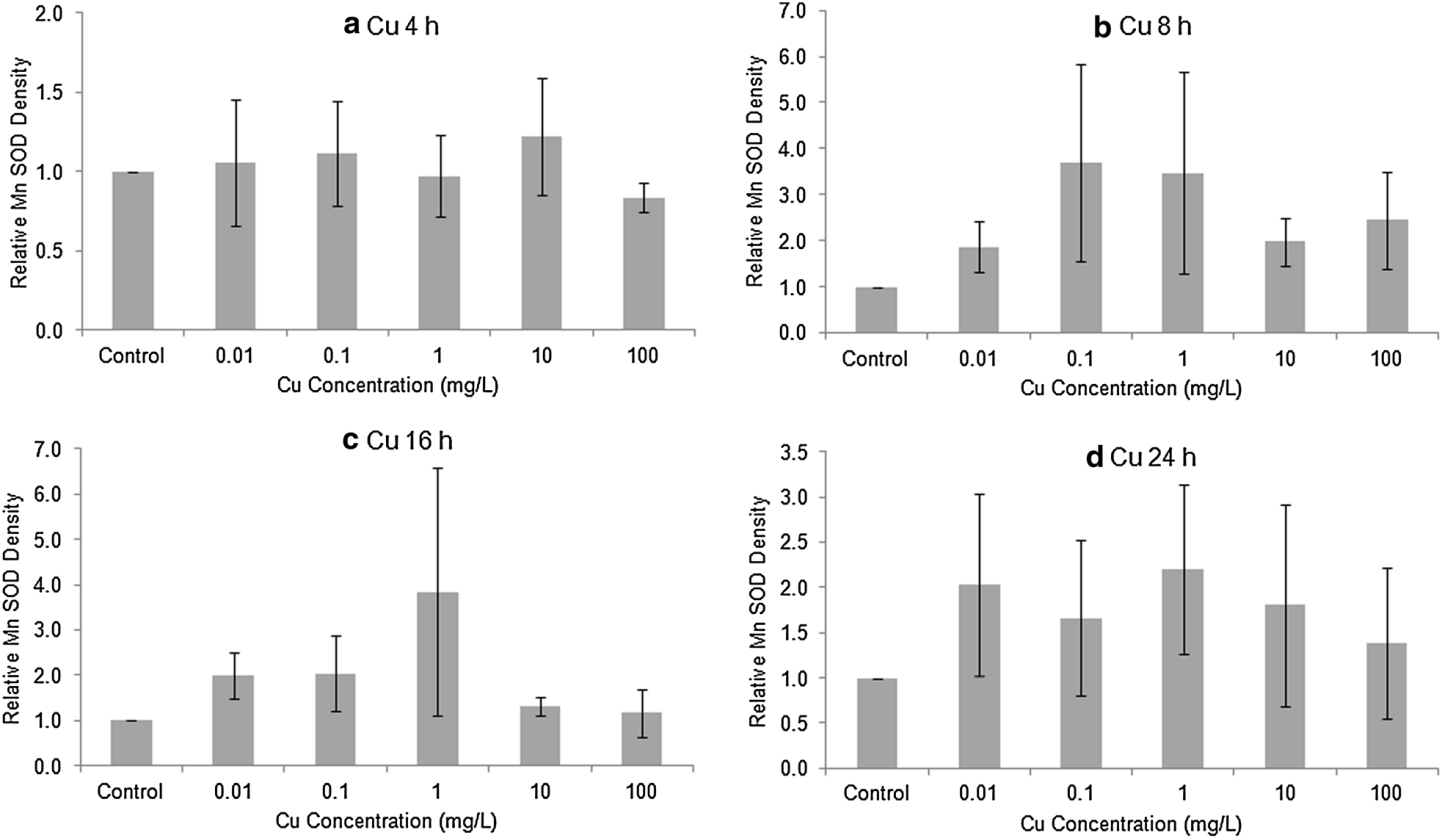
Relative amount of Mn SOD protein in Stage 20 *Limulus polyphemus* embryos exposed to Cu as determined by Western blotting. Data expressed as the mean ratio (±SE) of Mn SOD in exposed animals relative to seawater controls (n = 2–4 replicate determinations). **a** 4 h, **b** 8 h, **c** 16 h, and **d** 24 h acute exposure to Cu

**Fig. 5:**
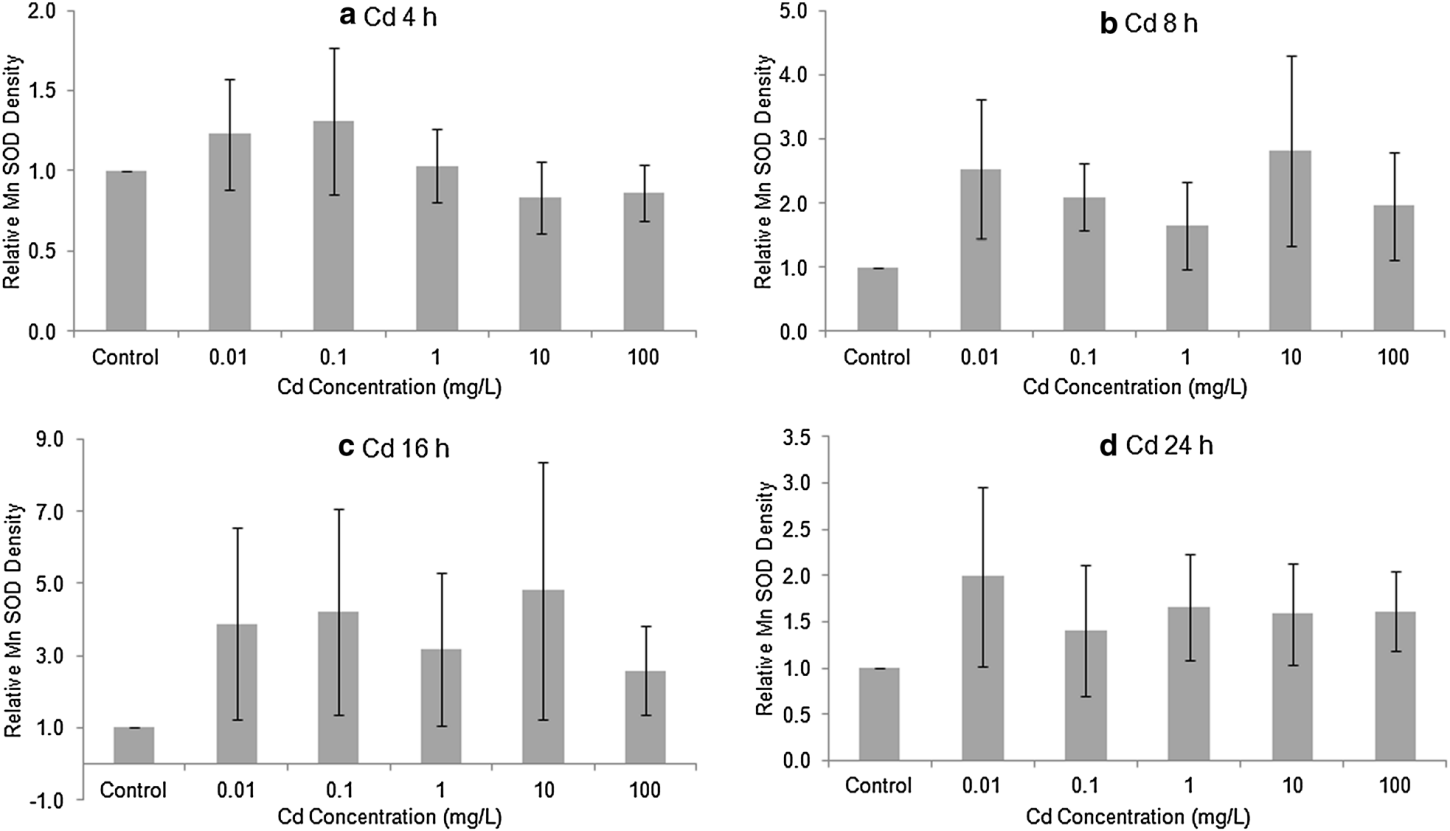
Relative amount of Mn SOD protein in Stage 20 *Limulus polyphemus* embryos exposed to as determined by Western blotting. Data expressed as the mean ratio (±SE) of Mn SOD in exposed animals relative to seawater controls (n = 2–4 replicate determinations). **a** 4 h, **b** 8 h, **c** 16 h, and **d** 24 h acute exposure to Cd

The level of Cu/Zn (cytosolic) SOD in Stage 20 embryos, as determined by Western blotting, was generally elevated after 4 or 8 h exposure to Cu or Cd (Figs. 2, 3). After 16 and 24 h exposure to Cu or Cd, the differences among treatments did not follow any consistent trend. To investigate whether there was any overall treatment effect, we pooled all four exposure times to compare against the controls. The high variability among replicates made the treatment differences non-significant for both Cu (1-way ANOVA, F = 1.194, 59 *df*, *p* > 0.32) and Cd (F = 0.419, 59 *df*, *p* > 0.80). The levels of Mn SOD in Cu or Cd treated embryos tended to be higher than in controls (Figs. 4, 5), but there was no overall significant treatment effect of either Cu (F = 1.137, 89 *df*, *p* > 0.34) or Cd (F = 0.744, 89 *df*, *p* > 0.59).

**Fig. 6:**
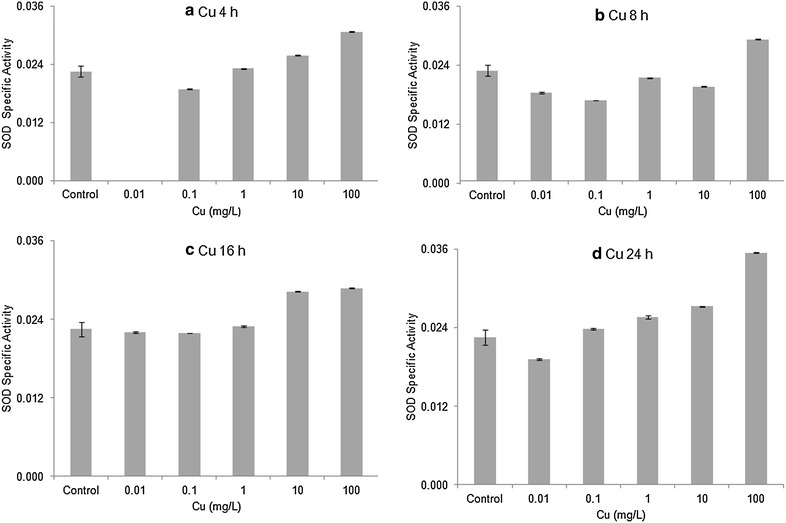
Enzymatic activity of SOD’s (mean ± SE) in Stage 20 *Limulus polyphemus* embryos exposed to Cu. **a** 4 h, **b** 8 h, **c** 16 h, and **d** 24 h acute exposure to Cu

**Fig. 7:**
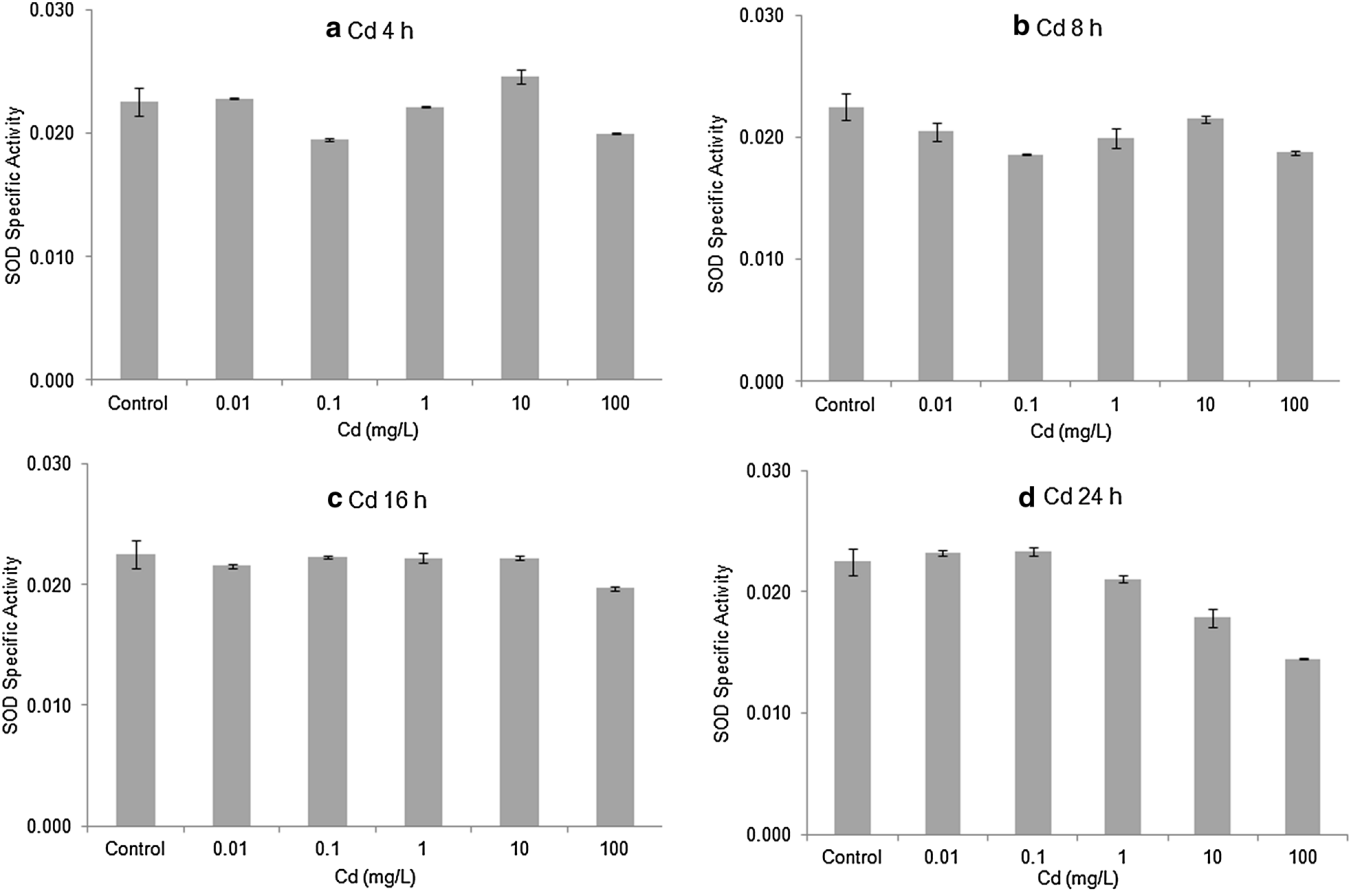
Enzymatic activity of SOD’s (mean ± SE) in Stage 20 *Limulus polyphemus* embryos exposed to Cd. **a** 4 h, **b** 8 h, **c** 16 h, and **d** 24 h acute exposure to Cd

Horseshoe crab embryos exposed to 10 and 100 mg/L Cu had the highest levels of SOD activity among all treatments (except for 10 mg/L at 8 h), but the differences between treatments and seawater controls were small. Overall, the enzyme activity of SOD showed no significant differences (ANOVA, *p* > 0.20 or higher) comparing embryos exposed to copper with seawater controls for any of the four time periods (Fig. 6). Similarly, there were no statistically significant differences in SOD enzyme activity (ANOVA, *p* > 0.17 or higher) comparing embryos exposed to cadmium with seawater controls in each of the four exposure times (Fig. 7). Table 2 shows the effects of the metals on the relative amounts of the two SODs. High values for the ratio mean decreases in the Mn SOD. The data for the copper series show more variability than do the Cd data. For Cd, the longer period of exposure to increasing concentrations of Cd seems have resulted in decreased amounts of Mn SOD.

**Table 2:** Ratio of Cu/Zn SOD to Mn SOD from enzyme activity before and after addition of NaCN which inhibits the Cu/Zn SOD

Time (h)	Cu concentration (mg/L)						Cd concentration (mg/L)					
	0	0.01	0.1	1	10	100	0	0.01	0.1	1	10	100
4	nd	nd	1.8	1.6	1.7	0.1	nd	0.008	0.008	0.008	0.010	0.007
8	nd	0.8	0.9	1.1	0.7	1.1	nd	0.62	0.74	0.77	0.84	0.58
16	1.7	2.4	2.0	1.8	1.7	0.3	1.65	0.62	0.74	0.77	0.84	0.58
24	1.9	2.3	0.5	1.6	1.3	0.2	1.92	0.89	1.42	1.20	1.61	2.26

* nd* No data

## Discussion

Stage 20 embryos of the American horseshoe crab had no significant mortality when exposed to CdCl_2_ at concentrations between 0.01 and 100 mg/L for up to 24 h (Table 1). Embryos exhibited little or no mortality when exposed to CuSO_4_ at concentrations of 0.01–10 mg/L for 4, 8, 16 or 24 h, although there was greater mortality at 100 mg/L. However, sublethal metal exposures prolonged development, leading to delayed hatching into the first instar larval stage (Fig. 1). These results are consistent with previous studies indicating that early stage horseshoe crabs are highly tolerant to pollution by metals and other contaminants, in comparison to the eggs and larvae of other marine invertebrates (Botton et al. 1998a, b; Botton 2000; Botton and Itow 2009). The delayed development observed in our study is consistent with previous work showing prolonged development in *Limulus* embryos exposed to tributyltin (Botton et al. 1998b), oil (Strobel and Brenowitz 1981), or subjected to osmotic stress (Greene et al. 2011). Additionally, Itow et al. (1998a) found that exposure of *Limulus* embryos to sublethal levels of Cu, Cd and other metals increased the frequency of developmental abnormalities, while also impairing the ability of small juveniles to regenerate appendages (Itow et al. 1998b).

It is well established that levels of ROS may become elevated in the presence of heavy metals and other environmental pollutants, and that excessive amounts of ROS can damage macromolecules including lipids and proteins (Lesser 2006; Lushchak et al. 2011). SOD is known to be an important part of the enzymatic antioxidant systems in aquatic arthropods (Fanjul-Moles and Gonsebatt 2011). However, investigations of either the Cu/Zn (cytosolic) or Mn (mitochondrial) forms of this enzyme in horseshoe crabs (which are chelicerates, not crustaceans) are lacking, aside from a report by Ding et al. (2005) that showed upregulation of Cu SOD, along with other stress-related enzymes including Hsp’s 90, 70, and 40 as part of the response to excessive ROS when mangrove horseshoe crabs (*Carcinoscorpius rotundicauda*) were infected with Gram-negative bacteria (*Pseudomonas aeruginosa*). SOD’s have been found in other chelicerate arthropods including mites (Zhang et al. 2014) and ticks (Ibrahim et al. 2013); the latter authors identified Mn and two different Cu/Zn forms of the enzyme.

Levels of both Cu/Zn SOD and Mn SOD tended to be slightly elevated in horseshoe crab embryos exposed to sublethal levels CdCl_2_ or CuSO_4_ (Figs. 2, 3, 4, 5). However, there was no clear relationship between metal concentration and amount of SOD in treated embryos, and no overall statistically significant treatment effect. Similarly, embryos exposed to Cu and Cd generally showed slightly higher specific activity of SOD’s compared with seawater controls, although once again, there did not appear to be a dose–response relationship and differences between treatments were non-significant (Figs. 6, 7). The lack of a significant increase in either SOD levels or specific activity differs from a number of other studies with other aquatic animals. Increases in SOD and other antioxidant molecules are commonly reported from crustaceans and other aquatic invertebrates exposed to pollution stress (Fanjul-Moles and Gonsebatt 2011). For example, Lyu et al. (2013) found significant increases in Cu/Zn SOD transcription and in SOD enzymatic activity in the cladoceran *Daphnia magna* after 48 h exposure to 5 μg/L CuCl_2_. When the polychaete, *Neanthes succinea,* was exposed to 12–72 μg/L CuCl_2_, Rhee et al. (2011) found increased expression of both the Cu/Zn and Mn SOD genes. SOD activity in the digestive gland of the bivalve, *Mactra veneriformis*, was significantly elevated following 3-day exposure to 50–200 μg/L CdCl_2_, although differences between Cd-exposed animals and controls were less distinct after longer exposure periods (Fang et al. 2010). SOD activity was significantly increased after Cd exposure in the freshwater crab *Sinopotamon yangtsekiense* (Lei et al. 2011). Geret et al. (2002) noted short-term increases in the activity of both cytosolic and mitochondrial SOD in clams (*Ruditapes decussatus*) exposed to 4–100 μg/L Cd. There was a significant increase in the SOD activity in shrimp (*Penaeus indicus*) post-larvae in following 24 h to 10 days of sublethal (0.16 ppm) copper exposure (Paila and Yallapragada 2011). In a field study, SOD and catalase activity were higher among mussels (*Mytilus galloprovincialis*) living at sites that had the highest in heavy metal concentrations (Vlahogianni et al. 2007).

The origin of the MnSOD that we detected in the “cytoplasm” (i.e., the 10 k RPM supernatant) is not clear, and may be due to lysis of mitochondria during the bead beating process. Or, it may be that Stage 20 embryos, which are between molts, have a “cytoplasmic” MnSOD as was detected in intermolt decapod crustacea by Brouwer et al. (1977, 2003). They hypothesize that the source of Cu for Cu/Zn SOD changes with the hemocyanin that may provide Cu utilized for Cu/Zn SOD. Stage 20 embryos do contain hemocyanin, but it is not the major protein (Hamilton, unpublished results). In any case, the ratios show decreases in the amount of MnSOD compared to the control for both Cu and Cd most dramatically for the 24 h samples. Attributing the presence of MnSOD in the cytoplasm to lysis may account for the delay in hatching (Table 1).

This research extends our understanding of the biochemical adaptations that facilitate enable horseshoe crab embryos to survive in stressful environments (Botton et al. 2006a, 2010; Greene et al. 2011). These mechanisms, notably the stress proteins HSP70 and HSP90, enable horseshoe crab embryos to survive pollution found in many estuarine spawning environments. Previous work has suggested that constitutive and/or inducible stress proteins are related to the ability of *Limulus* embryos to survive temperature and osmotic stresses (Botton et al. 2006a; Greene et al. 2011). To some degree, not yet investigated, the chorion and inner egg membranes (Sekiguchi et al. 1988; Botton et al. 2010) may act as barriers that prevent pollutants from the water or sediments from entering the embryos.

Collectively, our results demonstrated that horseshoe crab embryos exposed to sublethal levels of Cu or Cd exhibited signs of stress (as shown by delayed development), but neither the quantity or specific activity of SOD were significantly elevated in these stressed individuals. Thus, the protective role of SOD’s against ROS produced in response to metal exposure appears to be limited, at least under our experimental conditions. It is possible that longer exposure periods, higher pollutant loads, and/or different stressors would lead to quantitatively larger differences in SOD levels and specific activity between controls and treatments. In marine crustaceans, cellular damage from heavy metals may also be modulated through binding by metallothioneins, sequestration in metal-rich vacuoles, and other mechanisms (Ahearn et al. 2004), but these have not yet been studied in horseshoe crabs. Future research should investigate other potential mechanisms to protect against damage by ROS, including other enzymes such as catalase and peroxidases, and cytosolic scavengers molecules such as ascorbic acid, tocopherol, glutathione, and carotenoids (Lesser 2006; Fanjul-Moles and Gonsebatt 2011; Regoli and Giuliani 2014).

## Conclusions

In this study, we examined the physiological responses of horseshoe crab (*Limulus polyphemus*) embryos to short-term (4–24 h) exposure to Cu and Cd (0.01–100 mg/L). The percentage of embryos that survived and hatched into first instar larvae was generally unaffected by metal exposure except at the highest Cu concentration, but there were significant delays in developmental rate. Exposure to metals is believed to induce oxidative stress, but the quantity of cytosolic and mitochondrial SOD’s, as determined using Western blotting, were not significantly elevated in metal-exposed embryos. Similarly, there were no significant increases in the specific activity of SOD in response to metal exposure. We conclude that the protective role of SOD’s against metal exposure appears to be limited, least under our experimental conditions.

## Methods

### Collection and maintenance of eggs and embryos

Horseshoe crab eggs were collected from West Haven, Connecticut (Long Island Sound) and were reared at room temperature in sediment-free glass dishes at a density of approximately 500 eggs per dish. Artificial seawater (ASW) at 20 PSU was made using Instant Ocean™ in deionized water. ASW was refreshed daily, except over weekends. No food was provided because horseshoe crabs do not begin to feed until the second instar (“first tailed”) stage (Botton et al. 2010). We observed the embryos under a dissecting scope for staging. All experiments used Stage 20-1 embryos, the developmental stage where the third embryonic molt has been completed, the embryo has emerged from the outer egg membrane or chorion, and is now enveloped by the clear inner egg membrane (Sekiguchi et al. 1988). At this stage, the legs are segmented and the book gills and lateral eyes are distinct. Embryos for each experiment were separated into plastic Petri dishes with 125 individuals per dish, which were later divided randomly into separate groups for viability and developmental rate experiments and SOD assays (see below).

Stock solutions of 100 mg/L CuSO_4_ (Fisher Scientific) and CdCl_2_ (Aldrich Chemical Company) were made in ASW, and further dilutions were made in ASW to achieve nominal concentrations of 0.01, 0.1, 1.0, and 10.0 mg/L, with ASW as the control group. For each metal concentration, there were four exposure times of 4, 8, 16, and 24 h.

### Viability and developmental rate

Following exposure to Cu or Cd, embryos were washed twice with deionized water to remove residual metal solution, and 50 embryos from each treatment were cultured in plastic Petri dishes in 20 PSU ASWThe endpoint for viability was deemed to be hatching into the first instar (trilobite) larval stage. On each date that the dishes were inspected, live trilobite larvae were removed and the ASW was changed. Embryos that failed to hatch and showed no evidence of gill or leg movements were considered to be dead. Differences in mean date of hatching between ASW controls and metal treated groups were analyzed using the Student’s t test (α = 0.05).

### Preparatory procedures

Stage 20 embryos (75 per treatment) were exposed to nominal Cu and Cd concentrations of 0.01, 0.1, 1.0, 10.0, and 100.0 mg/L, with ASW controls, for periods of 4, 8, 16, and 24 h. Immediately after the conclusion of each trial, proteins were extracted in Tissue Protein Extraction Reagent (T-PER, Thermo Scientific) which contains a non-ionic detergent at pH 7. The embryos were divided into three 2-mL Bead Beater tubes (25 eggs per tube). The tubes were “bead-beat” in a FastPrep-24 instrument (MP Biomedicals) at 4 m/s for 60 s, and then centrifuged at 14,000 rpm for 10 min at 4 °C to remove the granite sand and cellular fragments. The 10 k rpm supernatants were stored at 4 °C until analysis.

Protein concentration for each treatment (i.e., each exposure time × metal concentration) was determined using the Pierce 660 nm Protein Assay Reagent (Thermo Scientific).

### Western blotting and immunodetection of SOD

We ran SDS-PAGE gels (gradient 4–20 %) in HEPES-SDS (NuSep Inc. North America). Volumes of samples were loaded with the intent of standardizing protein mass to facilitate quantitation. Actin was used as a loading control when possible. Proteins were transferred to PDVF membranes via electroblotting using Bio-Rad’s vertical system. Membranes were stained for total protein with AMRESCO ProAct Membrane Stain and erased before proceeding to immunodetection by the indirect method. The main supplier of primary antibodies in both rabbit and mouse hosts was Abcam (Cambridge, MA, USA). There was considerable variation in the “titer” of the antibodies. Therefore, it was not possible to obtain data on the relative amounts of each type of SOD from the Western blots (such estimates were made from the relative enzyme activity as described below). The secondary antibodies (LI-COR^®^ Biosciences) were detected and scanned with the Odyssey Infrared Imaging System at 680 nm (red) and 800 nm (green). Band intensity was quantified using UN-SCAN-IT Version 6.1 (Silk Scientific). Differences in band density among treatments were examined using 1-way ANOVA (α = 0.05).

### SOD activity

The combined enzymatic activity of both Cu/Zn SOD and MnSOD was determined using the SOD Assay kit-WST (Dojindo Molecular Technologies, Inc.). This kit provides a reagent for the assay of SOD enzyme activity that replaces cytochrome c in the procedure of Crapo et al. (1978). To detect changes in activity rather than units of SOD, the data in this paper are expressed as the ratio of the SOD activity to the measured total protein concentration in each sample, as averaged over three replicate determinations. The assay was repeated on samples treated with 1 mM NaCN which inhibits the Cu/Zn SOD to obtain the Mn SOD activity and the Cu/Zn SOD enzyme activity by subtraction. Differences among treatments in enzyme activity were examined using 1-way ANOVA (α = 0.05).
